# Oral health policy model for Turkey: how to deliver preventive services?

**DOI:** 10.3389/frhs.2025.1513688

**Published:** 2025-07-22

**Authors:** Ayşegül Doğan, Serap Durukan Köse

**Affiliations:** ^1^ Ahmet Erdoğan Vocational School of Health Sciences, Department of Dentistry Services, Zonguldak Bulent Ecevit University, Zonguldak, Türkiye; ^2^ Faculty of Health Sciences, Department of Health Management, Mugla Sıtkı Kocman University, Mugla, Türkiye

**Keywords:** oral and dental health, health policy, public oral and dental healthcare services, preventive oral health services, public health policy

## Abstract

**Background:**

Oral health is considered a neglected area in Turkey. To date, therapeutic services have dominated the provision of oral health care, while preventive services have been underfunded and inconsistently delivered. This study aims to elicit the need for the integration of preventive oral health services into the Turkish public health system by revealing the viewpoints of dentists, citizens, health managers and experts in Turkey.

**Methods:**

A snowball sampling method was used, and qualitative semi-structured interviews were conducted with 47 participants. Data were collected between April and August 2023. Both content and descriptive analyses were conducted, with content analysis performed using the MAXQDA 2023 software.

**Results:**

The codes were grouped into three main themes: opinions on the provision of preventive oral and dental health services, opinions about family dentistry, current problems in oral and dental health services in Turkey. Results indicate that there is an urgent need to provide preventive oral health services in a systematic way and that the public is unaware of oral health. Provision of oral health services within primary health care services should be presented through the family dentistry system.

**Conclusion:**

A roadmap for integrating preventive oral health services into the Turkish public health system was developed, incorporating preventive practices targeting both individuals and communities, using a public health approach.

## 1 Introduction

An individual's oral and dental health is a major factor in determining his or her wellbeing and quality of life (1). Most chronic oral diseases are preventable; however, their widespread prevalence positions them as a major global public health issue. Moreover, the high costs associated with their treatment impose a financial burden on societies, especially in low- and middle-income countries (2, 3). These diseases have detrimental effects on overall quality of life and have same risk factors with non-communicable diseases such as cancer, chronic respiratory diseases, and cardiovascular health situations (4, 5). Today, there is an increasing focus on improving oral health, promoting its development, preventing dental disorders before they arise, and intervening early (6).

The Ottawa Charter for Health Promotion, an international agreement organized by the World Health Organization (WHO) and signed in Ottawa, Canada, in November 1986, initiated a series of actions among international organizations, national governments and local communities to achieve the goal of “health for all”. The delivery of oral health services aligns with the broader movement in health services reform to guarantee primary health care and “health for everybody” (7). To improve oral and dental health, reduce the need for expensive treatments, and improve the general public's oral and dental health, many developed nations place a high priority on preventive care and provide it as a part of primary healthcare (8). To achieve this goal, national oral health policies have been created, public oral and dental health insurance plans been utilized, and macro-level public health programs have been conducted. However, there is a lack of adequate integration of oral health services into primary health care in Turkey. Treatment-oriented implementation comprises the majority of oral and dental health services. Services for oral health promotion and prevention are not routinely offered (9).

Prior to 2002, oral health services were primarily provided by private practitioners, often funded through out-of-pocket payments. The 2003 introduction of the Health Transformation Program marked a shift, enabling the Ministry of Health to significantly expand the scope and capacity of oral health services (10). Today, both public and private institutions provide these services (11, 12), though preventive care remains underfunded and inconsistently available (9). Oral diseases remain highly prevalent, and their treatment continues to be costly (13). Unlike many developed countries that have structured their oral healthcare systems around prevention, Turkey lacks a systematic approach to preventive oral care. The Family Dentistry model, introduced as a concept in 2004 to integrate dental services into primary care, was not implemented at the time (14). A pilot evaluation of this model was conducted in three provinces in August 2022. However, laws protecting and enhancing dental and oral health were put into place many years ago in most developed countries (15, 16). The growing global emphasis on prevention highlights the urgent need for Turkey to restructure its oral healthcare system around preventive principles. From a public health and health management perspective, determining where and how to deliver oral healthcare within the system is crucial. Preventive practices are especially emphasized in primary care, and global recommendations advocate for organizing oral health services accordingly (17).

A review of existing literature reveals a lack of practical models and a predominant focus on underlining the importance of prevention. This study seeks to identify the necessity of integrating preventive oral health services into Turkey's public health system by capturing the perspectives of dentists, citizens, health managers and specialists. It further aims to assess the current state of oral health services and propose a viable solution for incorporating preventive services into the primary healthcare infrastructure. are expected to support the development of a roadmap for the integration of preventive oral health services into the Turkish health system.

## 2 Materials and methods

A qualitative research method was employed to collect and analyze the data. The universe was categorized in the study's initial phase based on its specifics. A universe is intended to be created within the purview of the study of those who offer the service, those who use it, and those in charge of its administration and coordination. “Citizens” as service recipients, “dentists” as service providers, and “health politicians and experts” as accountable for service provision management and fees are the forms that comprise the universe. This group was assembled in order to conduct the snowball sampling method, which is regarded as one of the suitable sampling methods for qualitative research. Using the method, the researcher first identifies the most appropriate individuals by conducting interviews with the experts of subject matter (18). For the first interviews, dentists were selected from among those who accepted to participate in the research from dental professional chambers. The citizens group is limited to individuals who are over 18 years of age and have benefited from family medicine services at least once. The first interviews from the citizens group were initiated with citizens who met the required criteria. The first interviews from the health politicians and experts group were initiated with volunteers from the Provincial and District Health Directorates, Provincial Social Security Institution managers, family physicians and public health experts and then continued according to the snowball sampling process.

In the study, semi-structured interviews were used. Open-ended forms were produced for each participant group based on a thorough literature that the researcher did in compliance with the semi-structured interview technique. Interviews were terminated when thematic saturation was reached. A total of 47 individuals were included in the study. Of 12 participants were from the health politicians and experts group, of 15 participants were from the dentists group, and of 20 participants were from the citizens group. Both content analysis and descriptive analysis were used to analyse the data. Content analysis was performed by using the MAXQDA 2023 software package. In qualitative research, “reliability” is related to the honesty and integration of the researcher with the subject. Researchers spent time interacting with the participants for a long time and had the opportunity to closely examine their experiences and opinions. After each interview, the interview was summarized and participant confirmation was provided. Using the snowball sampling technique to reach the richest data sources on the subject played an important role in ensuring the validity and reliability of the study. To ensure intercoder reliability, coding was performed simultaneously by the researchers.

## 3 Results

Fifteen dentists were participated in the study. There were 7 male and 13 female citizens, three female managers, health experts and nine male managers. Demographic characteristics of the participants are shown in Table 1. Participants in the dentists group are indicated with D, citizens with C, and participants in the health managers and experts group are indicated with E. The codes produced as a consequence of content analysis were combined to generate categories and themes. The codes were gathered under three themes in the context of the study: opinions on the provision of preventive oral and dental healthcare services (ODHC's), opinions on family dentistry and current problems with oral and dental health services in Turkey. Assessing the views of Turkish citizens, dentists, politicians and experts on preventive oral health services is crucial for exposing the current state of affairs, pointing out shortcomings, and comprehending expectations.

**Table 1:** Demographic characteristics of participants.

Group	Participant	Age	Gender	Occupation
Health Managers and Experts	E1	44	M	Doctor
	E2	40	M	Provincial Health director
	E3	43	F	Public Health Expert
	E4	45	F	Provincial Health director
	E5	51	M	Social security institution manager
	E6	36	M	Public Health Expert
	E7	48	M	Provincial Health director
	E8	39	M	Head of provincial public health services department
	E9	44	M	District health director
	E10	47	M	District health director
	E11	53	M	Provincial Health director
	E12	56	F	Doctor
Dentists	D1	64	M	Private sector dentist
	D2	44	M	Private sector dentist
	D3	29	F	Public sector dentist
	D4	41	F	Private sector dentist
	D5	35	F	Public sector dentist
	D6	28	F	Public sector dentist
	D7	39	F	Public sector dentist
	D8	41	M	Public sector dentist
	D9	57	F	Private sector dentist
	D10	39	F	Private sector dentist
	D11	36	F	Private sector dentist
	D12	40	M	Public sector dentist
	D13	30	F	Private sector dentist
	D14	41	F	Public sector dentist
	D15	50	M	Private sector dentist
Citizens	C1	39	F	Teacher
	C2	31	M	Technician
	C3	34	M	Computer Engineer
	C4	35	M	Civil Engineer
	C5	29	F	Office Clerk
	C6	38	F	Saleswoman
	C7	42	M	Technician
	C8	28	F	Architect
	C9	32	F	Operator
	C10	44	F	Estate agent
	C11	29	E	Office Clerk
	C12	42	F	Saleswoman
	C13	40	F	Laborant
	C14	32	F	Teacher
	C15	39	F	Accountant
	C16	47	F	Accountant
	C17	30	M	Technician
	C18	59	F	Technician
	C19	32	M	Financial Advisor
	C20	45	M	Operator

**Figure 1:**
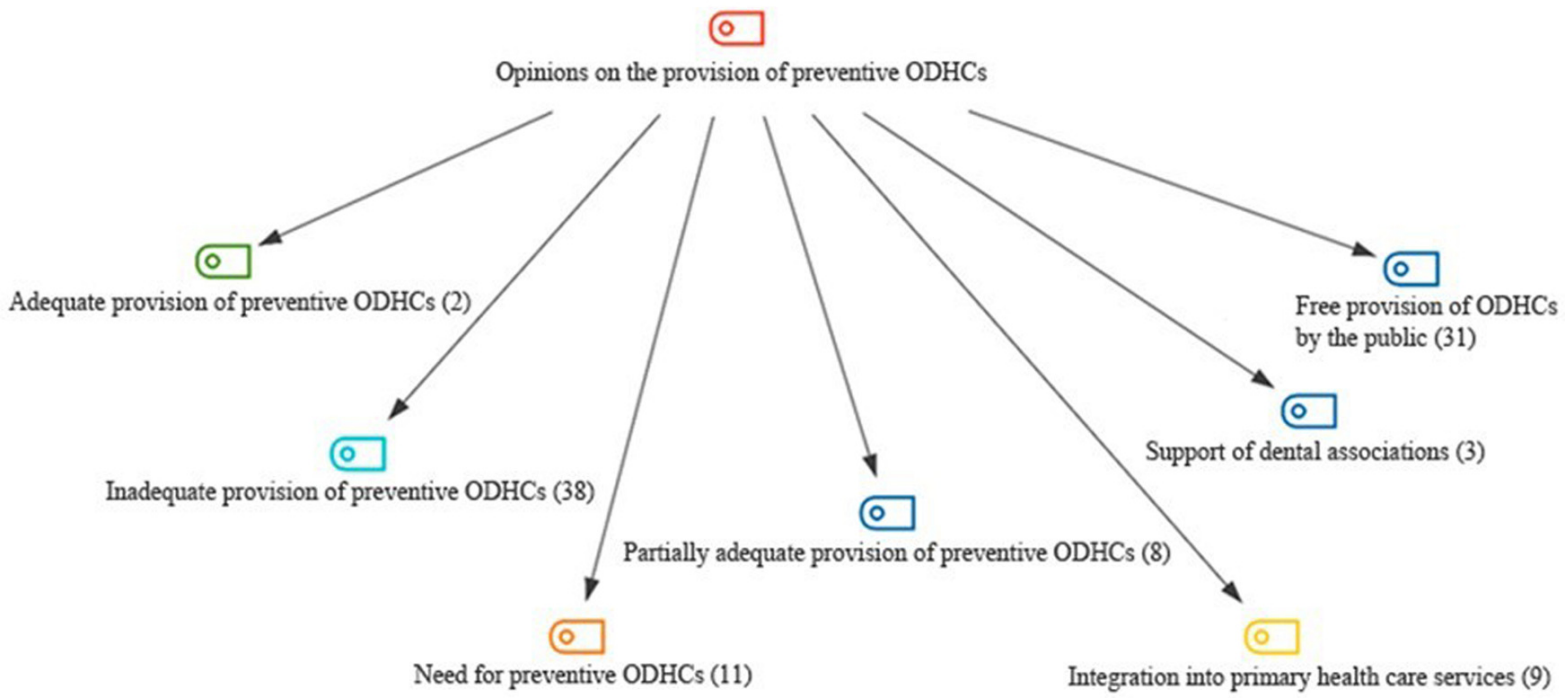
Opinions on the provision of preventive ODHC's; codes with frequencies.

Figure 1 shows the codes and frequencies formed under “Opinions on the provision of preventive oral and dental healthcare services(ODHC's)” theme. 7 codes have formed under this theme. The code with the highest frequency (f = 38) is “Inadequate Provision of Preventive ODHC's)”. “Adequate Provision of Preventive ODHC's” (f = 2) has the lowest code frequency. 

**Figure 2:**
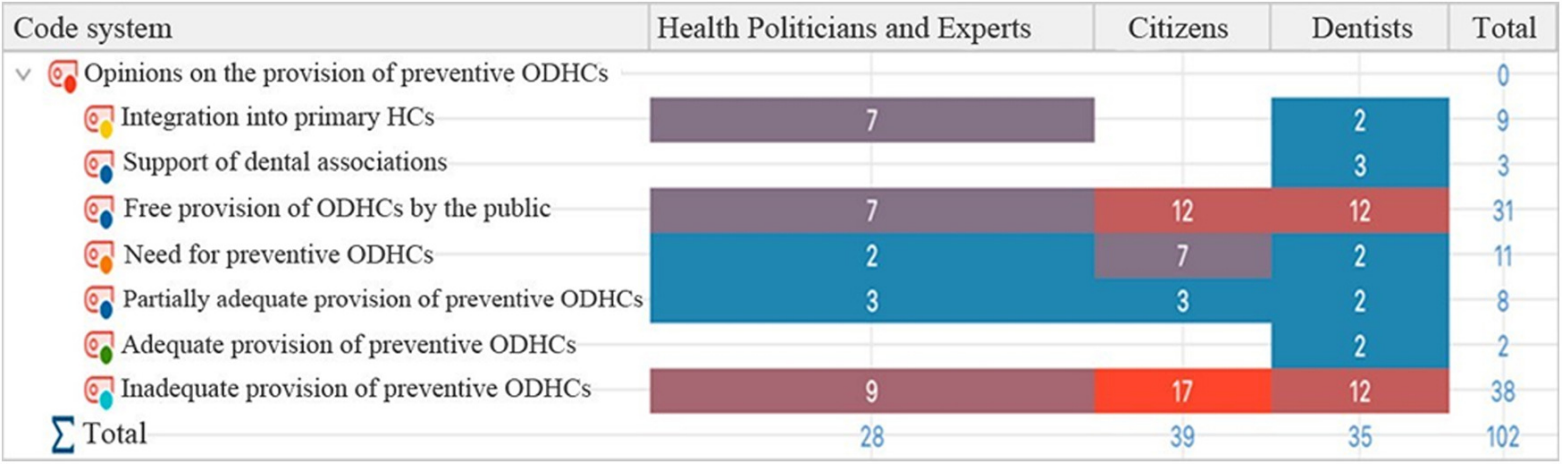
Opinions on the provision of preventive ODHC's; distribution of code frequencies by sample groups.

Figure 2 shows the distribution of code frequencies by sample groups. The codes with the highest frequency are “Inadequate Provision of Preventive ODHC's”(f = 38) and “Free provision of ODHC's by the public” (f = 31) for all groups. Among the panel of health managers and specialists, “Inadequate Provision of Preventive ODHC's”, “Free Provision of ODHC's by the public” and ‘Integration into primary HC's are the codes that are most frequently used. Most people have thought about Turkey's inadequate supply of preventative oral healthcare.

Some of the participants' opinions are given below:

“… *Preventive oral health services in Turkey are currently inadequate. School children cannot have sufficient access to treatment. Screenings and fissure sealants are positive in the first step, but there are deficiencies later on. It gets blocked somewhere, in other words, the incident does not continue. Like taking just one step and getting stuck on the road”* (D11)

“*.it is necessary for preventive oral health services to be more important. But for now, there is no dentistry at the primary healthcare in Turkey. Dental hospitals provide curative services, not preventive ones.”* (E6).

“*It should be mandatory for people to go for check-ups and have procedures that will protect their teeth, there is a great need for preventive services.”* (C12).

**Figure 3:**
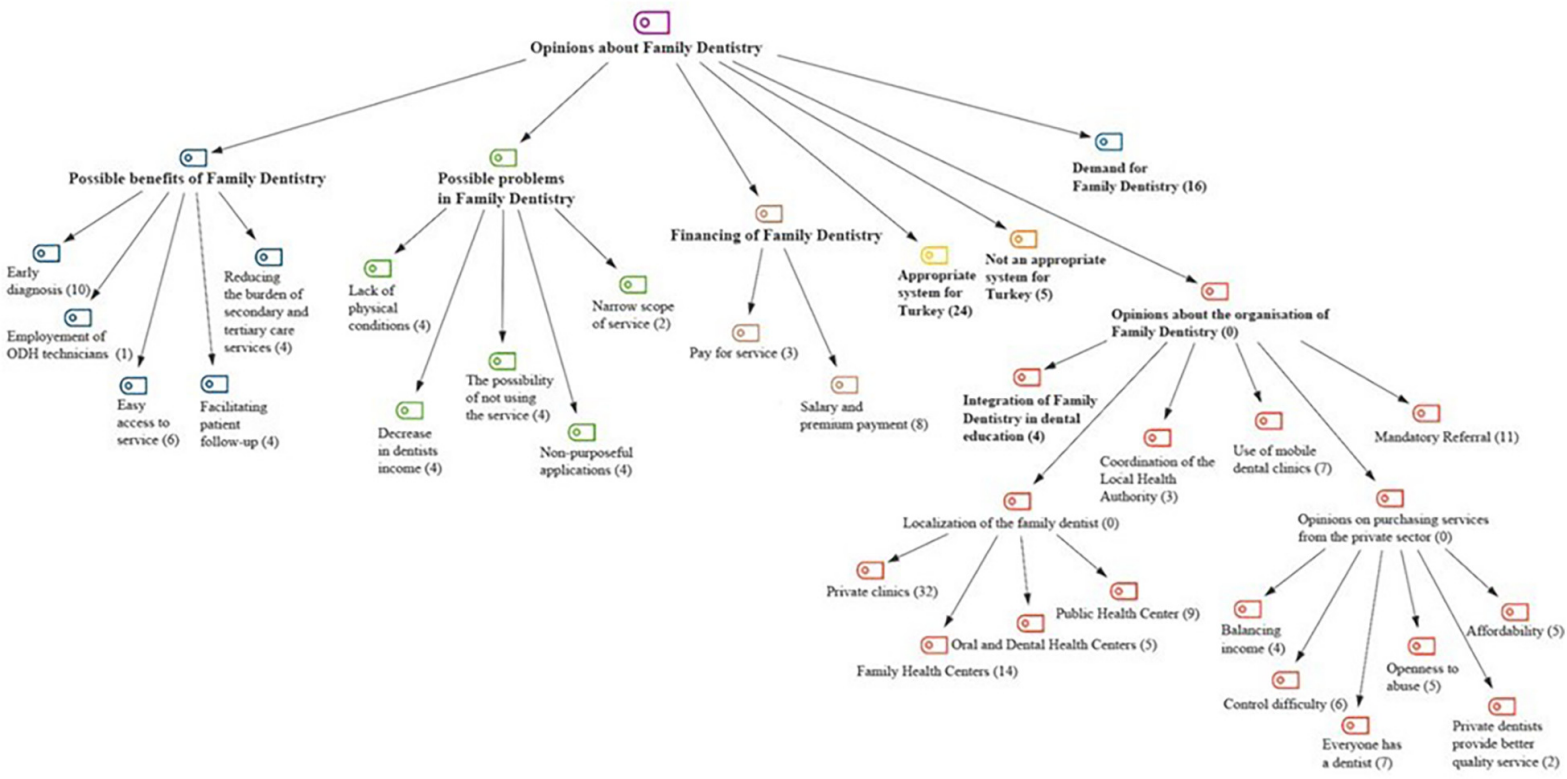
Opinions on family dentistry; categories with frequencies.

The second theme was the “opinions on family dentistry”. 7 categories were formed under this theme. Most participants think that family dentistry is an appropriate preventive oral health service delivery model for Turkey (f = 24) and it should be service should be provided in private clinics (f = 32). There is a demand for family dentistry (f = 16) but that it will only become efficient with a mandatory referral system (f = 11). Under this theme, 5 codes were generated under the category of possible benefits of family dentistry and 5 codes were generated under the category of possible problems that may be encountered in family dentistry (Figure 3).

**Figure 4:**
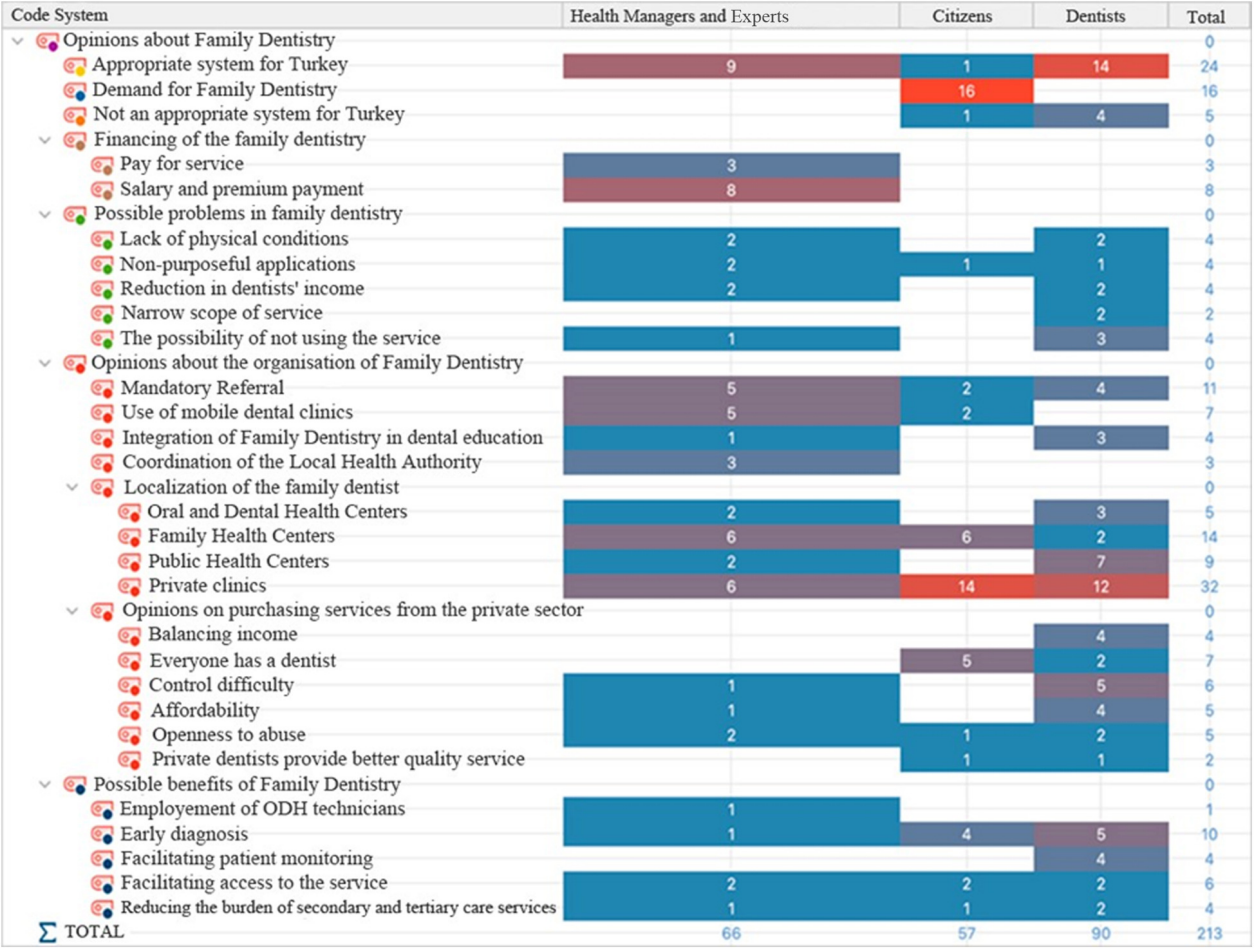
Opinions on family dentistry; distribution of code frequencies by sample groups.

Figure 4 shows the distribution of codes and categories based on sample groupings. The most emphasized issue in the health managers and experts group is that Family Dentistry is a suitable system for Turkey. In this group, the code named “appropriate system for Turkey” was coded 9 times by 12 participants. The most emphasized issue in the citizens group is that citizens want to have a family dentist. It was coded 16 times in total from the data of 16 out of 20 participants. In the dentists group, the most emphasized issue is that it is suitable for Turkey and that family dentistry should be provided in private clinics.

Some of the participants' opinions on the subject are given below:

“*I think it would be very beneficial if everyone had a family dentist. We would have easier access to dental services. I wish it had happened earlier in Türkiye. But I think our public health system's infrastructure is insufficient to implement this. “* (C1).

“*In our country, primary health care services are provided through the family medicine model, family dentistry system should also be integrated into this model. I believe it will be suitable for Türkiye. There is a serious need. Public and private cooperation should be established.”* (D2).

**Figure 5:**
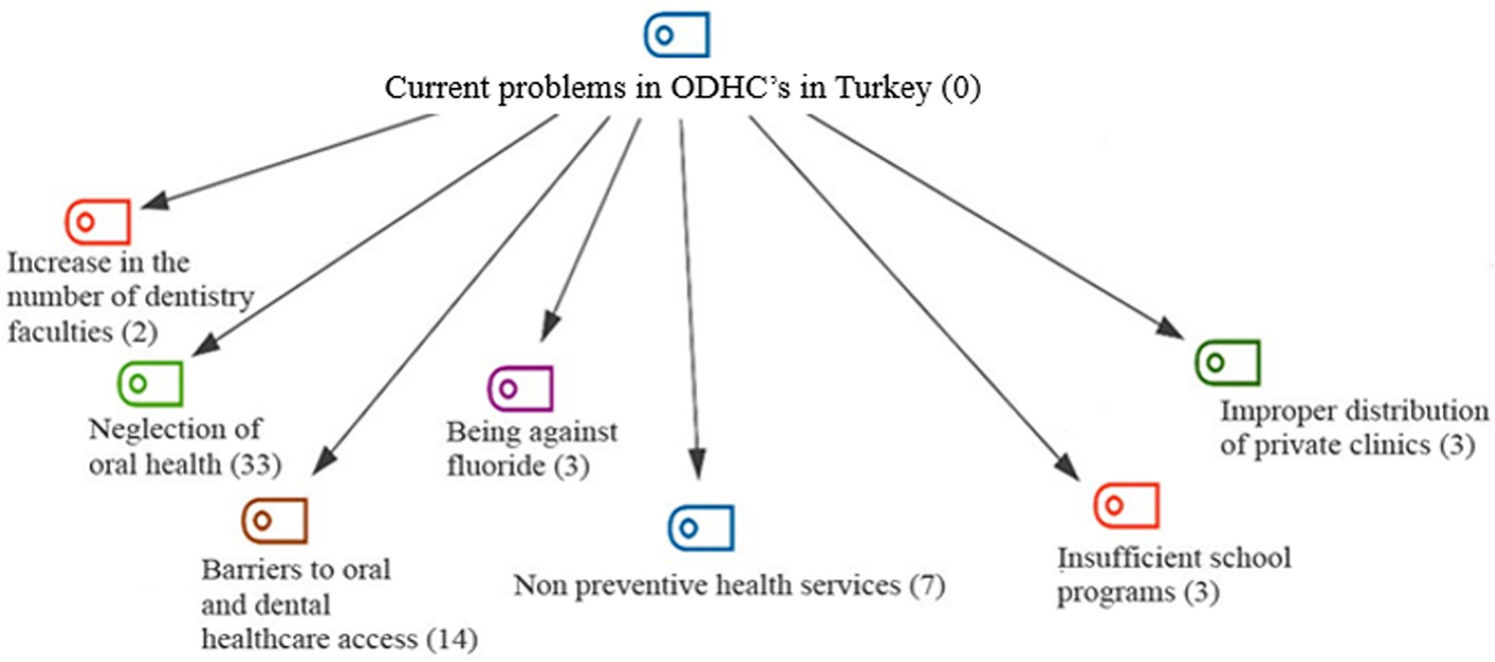
Current problems in oral and dental healthcare services in Turkey; codes with frequencies.

Under the theme named Current problems in ODHCs in Turkey, there were 7 codes formed. The most often occurring codes were those that referred to non-preventive healthcare services (*f* = 7), barriers to oral healthcare access (*f* = 14), and neglection of oral health (*f* = 33). All the codes are presented in Figure 5.

**Figure 6:**
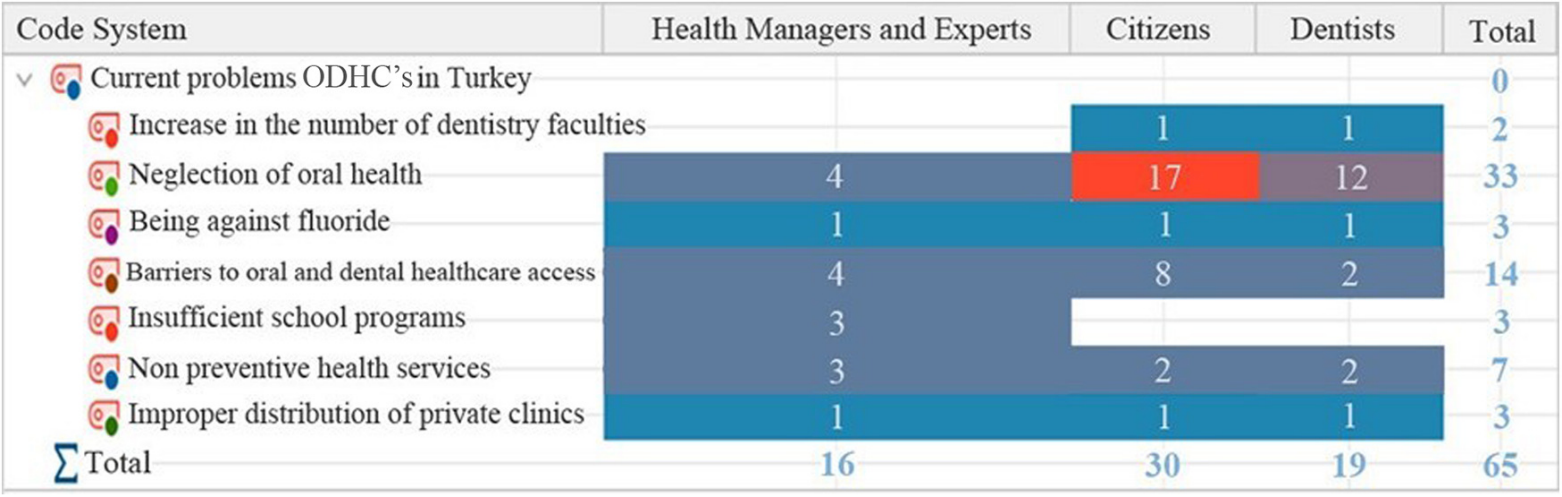
Current problems in oral and dental health services in Turkey; distribution of code frequencies by sample groups.

Upon analyzing the code distribution based on the sample groups it can be observed that “neglection of oral health” and “barriers to oral and dental health care access” are the most frequently occurring codes within the health managers and experts group. The code “neglection of oral health” is the most frequently occurring code among the citizen and dentist groups (Figure 6).

Some of the participants' opinions on the subject are given below:

“*People in Turkey ignore dental health. That's why tooth decay is so common and dental hospitals are overcrowded.”* (C3).

“*The number of dentistry faculties is increasing significantly, this is a big problem. I wonder if more qualified dentists are being trained; I don*’*t think so.”* (D15).

### 3.1 An oral health policy model for Turkey

Currently in Turkey, dental fluoridation is implemented only for school-aged children as a preventive measure. In addition, pit and fissure sealants are applied in clinics, but only upon patient request. There are no systematic preventive practices targeting other vulnerable groups such as the elderly, pregnant individuals, or those requiring home care. Preventive approaches should be delivered systematically and should encompass the entire target population (9). Based on the findings of this study, an oral health policy model has been proposed for Turkey. The key components of this proposed model for delivering preventive oral and dental healthcare include: incorporation into primary medical care, support of private sector, sustainability and equity, cost effectiveness, government funding, increasing societal consciousness, integration into home health services.

**Figure 7:**
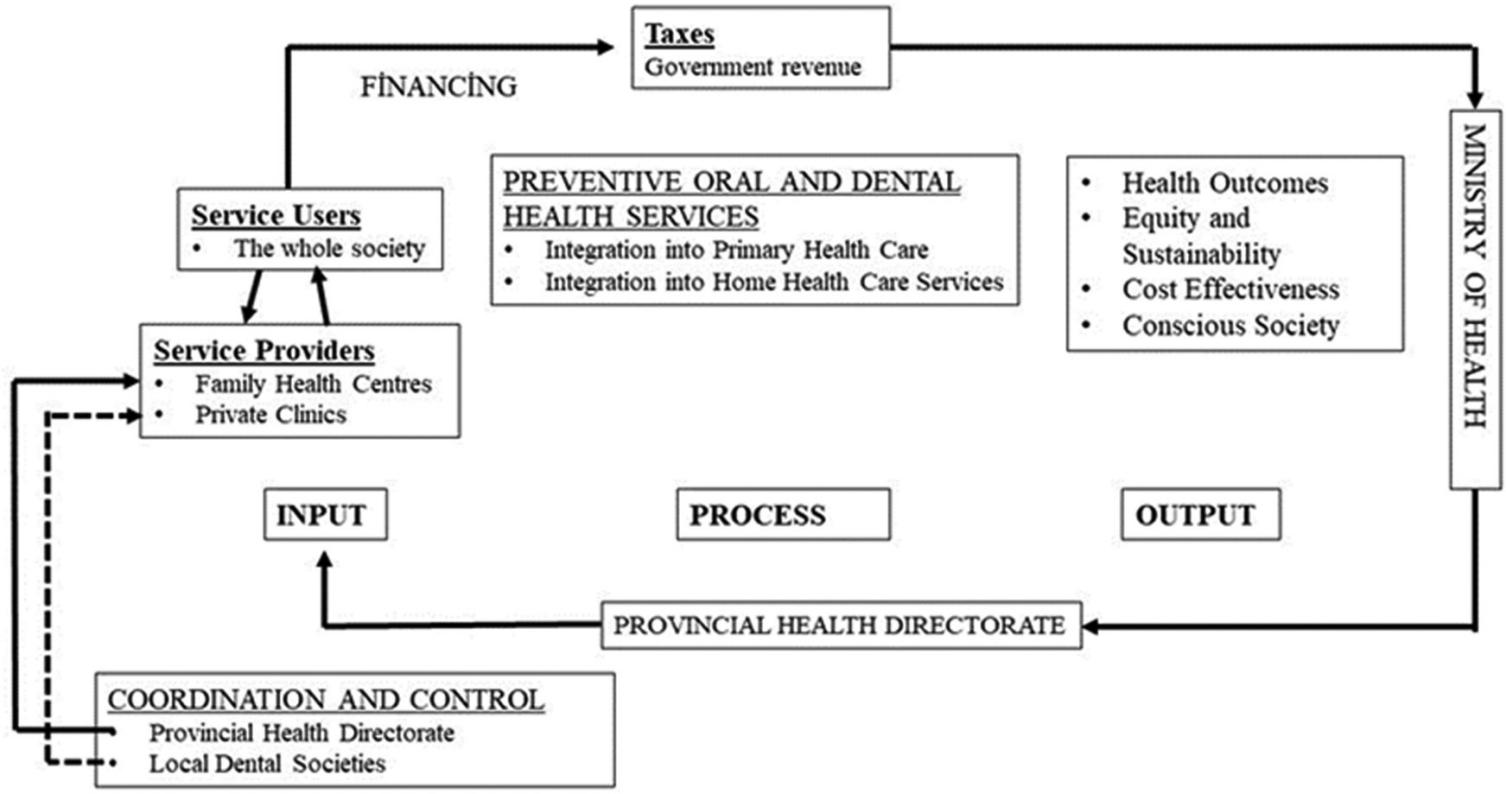
Oral health policy model for Turkey.

A collaborative effort between the public and private sectors is essential to enhance the integration of primary care with home health services. The model anticipates outcomes such as increased public awareness, equitable and sustainable service provision, and a reduction in the need for costly, advanced treatments. The proposed model for integrating preventive oral and dental health services into the Turkish public health system is illustrated in Figure 7.

Oral diseases should be addressed from a public health perspective. It is believed that by incorporating oral and dental health services within the primary health care packages, health disparities can be avoided and service accessibility will be improved. Turkey should adopt the Family Dentistry Model as a recognized model for incorporation into primary healthcare services. Family dentists can be localized in Family Health Centers (FHC) or private clinics. Family Health Center (FHC) family dentistry departments may be connected to the Provincial Health Directorate's Public Health Department. Therefore, Public Health Departments may create Family Dentistry Units.

The public ought to be the principal responsible player for preventive oral health services. Family dentists should offer preventative care at no cost to pregnant patients, people with certain chronic diseases, people with physical or mental disabilities, and anybody whose dental health significantly affects their overall health. The demand for oral healthcare should be financed by general funds. In this manner, financial resources should be directed by the Ministry of Health to the provincial health directorates and family dentists should get paid back.

The most cost-effective preventive strategy is to encourage individuals to take responsibility for their own oral hygiene. Achieving this requires comprehensive public education and awareness campaigns across Turkish society. Family dentists could organize oral hygiene education programs in kindergartens and primary schools, helping instill lifelong habits from an early age. In addition to children, teachers should also receive oral hygiene training. Elderly individuals, people with disabilities, and their caregivers should be educated about proper oral hygiene practices. As many in these groups experience difficulty accessing health facilities, home healthcare becomes crucial. For example, fluoride varnish can be applied at home to prevent tooth decay, and removable dentures can be adjusted or customized to prevent oral sores. Increasing the number of mobile dental vans can be viewed as a means of providing on-site care to individuals in remote areas who face challenges in reaching both homes and health facilities. While the Family Dentistry Model offers promise, the study also highlights potential challenges. Existing healthcare institutions may lack the infrastructure to support this new service. The limited technical capacity, narrow scope of services, patient resistance to using family dentistry in favor of hospitals, and complications related to referrals are identified as potential barriers to implementation.

## 4 Discussion

The primary aim of preventive oral and dental health services is to mitigate the onset of diseases by promoting proper oral hygiene, a balanced diet, and regular check-ups. Several factors—including individual characteristics, social determinants, and the structure of national health systems—affect the utilization of such preventive services. In countries where these services are not widely accessible, treatment costs and oral healthcare expenditures tend to be significantly higher (20). Most participants in this study believed that preventive oral health services are insufficiently provided in Turkey. Notably, many dentists emphasized the lack of adequate preventive care, which is a critical finding. Members of the health managers and experts group frequently underlined that such services should be integrated into the scope of primary healthcare. Globally, interdisciplinary collaboration within primary healthcare systems—including oral and dental health services—is known to enhance population health, prevent diseases, and reduce disparities in healthcare access (7). Primary healthcare centers around the needs of the community and addresses the consequences of chronic, non-communicable diseases (21).

Oral diseases are linked to numerous chronic conditions and have an impact on overall health and the entire body. Common modifiable risk factors for oral and chronic diseases include stress, smoking, and dietary patterns. Thus, it is imperative that primary health care services include provisions for the maintenance and improvement of oral health (19, 22). Ekici et al. (2017) found significant issues with the delivery of oral health care in Turkey. Of these issues, the most significant they claimed was the insufficient availability of preventative oral and dental health care (23). The results of our study align with these assumptions. Participants stressed the need for the public offering of preventive oral healthcare services at no cost. The private sector is known to be involved in the provision of preventive services, which are mostly provided by the public to children and young people aged 0–18 years for free in many countries (24).

Family dentistry was brought to Turkey's attention through the Health Transformation Project. Çam and Kumru (2019) conducted an evaluation of the views of patients and dentists on family dentistry. They found that 80.5% of the patients and 72% of the dentists agreed with the necessity of the family dentistry model (14). According to the results of our study, citizens demand family dentistry, and health managers and dentists think that this model is suitable for Turkey. Many countries have successfully adopted the family dentistry system and these initiatives have expanded access to oral and dental health services (25, 26). It's crucial to consider where family dentists should be situated. According to some participants, public family health centers should provide family dentistry services. Time would be saved if the family dentist and doctor were located in the same location, according to a few participants, particularly those from the citizens group. Most participants believed that family dentists in private practice should provide family dentistry. In the dentist group, this topic was brought up the most. Currently, oral health centers exist in significant districts and provinces where any person can obtain dental and oral care covered by insurance. However, public dental hospitals are overcrowded and unable to meet needs. Prasad et al. (2019) emphasized the need for both public and private sector support in order to provide oral and dental health services at the primary level, to increase access to services, and to be economically sustainable (22).

Our study's results highlight certain benefits, including the use of the system's current infrastructure and physical equipment, which will enable it to be developed more affordably and lessen income disparities across private dental clinics. In such a model, problems that may arise in the case of purchasing services, coordination and control problems may occur. Private clinics are concentrated in city centers where the population is high, and this may create a need for a different organization for rural areas. Preventive services can be rendered by mobile dental clinics, particularly to those in remote areas and those in need of home care. According to Ganavadiya et al. (2014), these mobile dental clinics equip people with disabilities to receive medical care outside of traditional healthcare settings (27).

Determining the current issues with Turkey's oral and dental health services is crucial since it serves as the foundation for future changes. The most commonly mentioned problem across all sample groups was the neglection of oral health. In line with these results, Topal et al. (2019) reported that Turkey's oral health indicators fall behind those of developed countries and that there is a deficiency in oral health awareness (28). According to Tokuç and Yıldırım's (2020) research, the lack of awareness in Turkey makes it difficult to effectively introduce tooth brushing practices to the society (29). Data from 2021 show that Turkey has a 0.4% annual dental visit frequency, compared to a 5% frequency in European countries (30). Every country incorporates oral health care into its health systems through the development of unique plans aimed at ensuring that these services are available to the whole population, preventing health disparities, and fostering lifetime dental and oral health (31).

This study is also subject to certain limitations. Firstly, snowball sampling may introduce sample bias. The perspectives gathered may not be representative of all regions and certain policymakers not included in the study. Additionally, the cross-sectional design limits the ability to assess the longitudinal impact of policy. The citizens group is limited to individuals who are over 18 years of age and have benefited from family medicine services at least once. Future research should include participants from more diverse regions and occupational groups.

## 5 Conclusion

Oral health is widely recognized as an integral part of overall health, as disorders in the oral cavity can affect not only the mouth but the entire body. The high prevalence and costly treatment of oral diseases impose a significant financial burden on national welfare systems. Therefore, implementing effective preventive measures has both economic and public health benefits. This study aimed to provide solutions for integrating preventive oral and dental health services into the Turkish public health system. It sought to present the views of dentists, citizens, health managers, and experts on the current state of oral healthcare in Turkey. The research comprehensively explored the optimal delivery methods for preventive services, highlighting deficiencies in the existing system and presenting an organized and evidence-based framework.

The most pressing issue identified in Turkey is the general lack of public awareness about oral health. As a result, there has been a noticeable increase in oral and dental diseases in society and disease treatments are prioritized to counteract this. The components of the model that is suggested to be created for the delivery of preventive oral health care in Turkey are incorporation into primary medical care, support of private sector, sustainability and equity, cost effectiveness, government funding, increasing societal consciousness, integration into home health services. With the implementation of the policy model proposed in this study, preventive oral health services can be integrated into the Turkish public health system. With this publicly funded system, individuals up to the age of 18 will be able to benefit from preventive oral health services free of charge. In addition, preventive services will be provided free of charge for individuals with physical and mental disabilities, pregnant women and individuals with chronic diseases. Home care services—previously transferred entirely to hospitals in 2017—would be incorporated into family dentistry units under this model. Future research should focus on testing the proposed model across diverse regions to assess its adaptability and effectiveness. Comparative analyses with existing health policy models could further clarify the strengths and potential areas for improvement in the suggested approach.

## Data Availability

The original contributions presented in the study are included in the article/Supplementary Material, further inquiries can be directed to the corresponding author.
